# Chronic mucocutaneous anogenital herpes: series of ten cases and literature review^☆^

**DOI:** 10.1016/j.abd.2021.03.014

**Published:** 2022-03-16

**Authors:** Karina Baruel de Camargo Votto Calbucci, John Verrinder Veasey

**Affiliations:** Dermatology Clinic, Santa Casa de Misericórdia de São Paulo, São Paulo, SP, Brazil

**Keywords:** Antiviral agents, Genital diseases, female, Genital diseases, male, Herpes genitalis, Herpes simplex

## Abstract

Anogenital herpes is one of the most prevalent sexually transmitted infections worldwide. It has several clinical manifestations, from cases of primary herpes infection to recurrent forms, among them chronic mucocutaneous herpes. This presentation is rare, characterized by a duration of more than four weeks, usually associated with immunosuppression and resistance to classic anti-herpetic treatment. The present study presents a series of ten cases of chronic mucocutaneous herpes with a discussion about its clinical, laboratory, and therapeutic aspects.

Chronic mucocutaneous herpes (CMH) is the infection caused by the herpes simplex viruses (HSV) type 1 and 2, lasting more than four weeks, resistant to classic anti-herpetic treatment.^1^

CMH has atypical clinical manifestations, requiring confirmation by a complementary cytological, laboratory, or anatomopathological method.^1^ In general, it is clinically characterized by painful, ulcerated lesions that show progressive growth, occasionally to verrucous, vegetative, or tumor forms, particularly in HIV immune reconstitution syndrome.^1^, ^2^ The treatment is challenging: the host usually has marked immunosuppression, which hinders the effective defense response against the offending agent and efficient wound healing and, on the other hand, intrinsic viral resistance to classic drugs such as acyclovir and valacyclovir has been increasingly described.^3^, ^4^, ^5^, ^6^

Ten patients with clinical and laboratory diagnoses of CMH were included in a retrospective study in the Dermatology Clinic of a tertiary hospital in São Paulo, Brazil, between January 2013 and November 2020. Detailed data are shown in Table 1 and clinical aspects in Figure 1, Figure 2. It is emphasized that all patients reported a history of local herpetic disease, indicating that the CMH forms would be variants of recurrent herpes and not of primary infection.

**Table 1:** Chronic mucocutaneous herpes: clinical aspects of the ten patients evaluated, methods and therapeutic response.

Characteristics of the patients				Characteristics of the lesions		Diagnosis		Treatment			
Case	Age	Sex	Comorbidities	Time of lesion	Location	Tzanck test	Biopsy (IHC)	Antiviral	Via (VO/EV)	Daily dose	Time until cure
1	15	M	Type 1 Diabetes mellitus	3 months	Genital	NP	NP	Acyclovir	PO	1,200 mg	4 weeks
2	52	F	HIV (CD4 430 cells/mm^3^)	1 month	Genital	NP	+	Valacyclovir	PO	1,000 mg	10 weeks (+ surgery)
3	51	F	HIV (CD4 6 cells/mm^3^)	1 month	Genital	+	NP	Acyclovir	IV	2,400 mg	4 weeks
4	15	F	Chronic mucocutaneous candidiasis	1 month	Gluteus	+	NP	Valacyclovir	PO	1,000 mg	6 weeks
5	59	M	Albinism	1 month	Genital	+	NP	Acyclovir	PO	4,000 mg	8 weeks
6	46	M	Alcohol abuse	1 month	Genital	+	NP	Acyclovir	PO	1,200 mg	6 weeks
7	58	M	HIV (CD4 187 cels/mm^3^)	18 months	Genital	NP	+	Acyclovir	PO	4,000 mg	Abandoned treatment
8	30	F	HIV (CD4 128 cells/mm^3^)	3 months	Anal	+	NP	Acyclovir	IV	2,400 mg	Death
9	41	M	HIV (CD4 112 cells/mm^3^)	9 months	Genital	+	–	Acyclovir	VO	1,000 mg	Abandoned treatment
10	30	F	HIV (CD4 51 cells/mm^3^)	3 months	Genital	NP	+	Foscarnet	IV	5,280mg	3 weeks

M, Male; F, Female; NP, Not performed; +, Positive; −, Negative; HIV, Human Immunodeficiency Virus; PO, Oral route; IV, Intravenous route.

**Figure 1:**
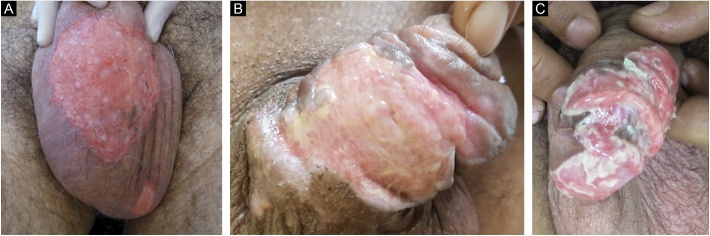
Chronic mucocutaneous herpes: clinical images of three male patients included in the study (A, Case 7; B, Case 6; C, Case 9).

**Figure 2:**
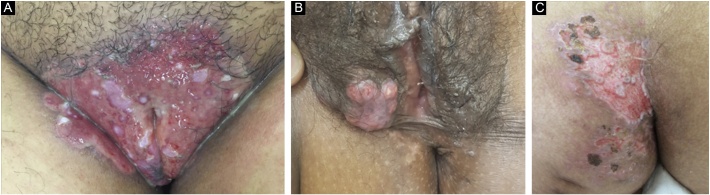
Chronic mucocutaneous herpes: clinical images of three female patients with genital and gluteal lesions (A, Case 3; B, Case 2; C, Case 4).

Anogenital cases of HSV-2 have up to six times more episodes of recurrence than those attributed to HSV-1, in addition to manifesting in a subclinical form in 10%‒25% of patients.^7^ Tzanck test (Fig. 3) and immunohistochemistry for HSV1 + 2 do not differentiate between parasitism resulting from HSV1 or HSV2. The diagnosis of CMH cases should be performed preferably with a skin biopsy, as the sample can also be analyzed by *in situ* hybridization and polymerase chain polymerase chain reaction (PCR aiming at viral identification.^3^, ^4^, ^8^ Moreover, the test rules out differential diagnoses of genital ulcers such as syphilis, cytomegalovirus infection, chancroid, fungal or protozoal infections.

**Figure 3:**
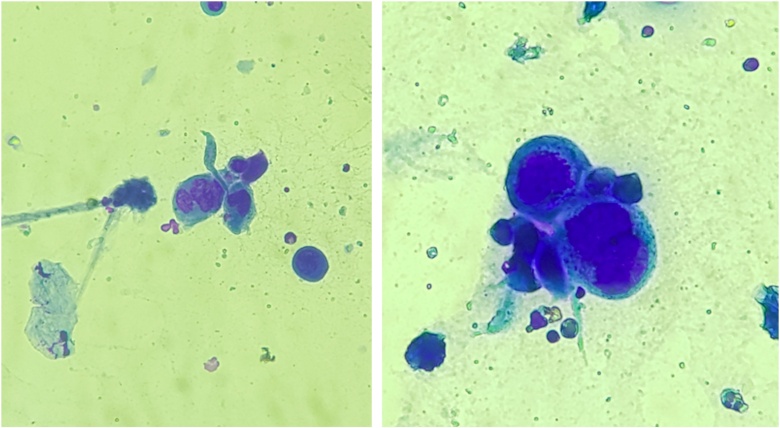
: Smear of lesions submitted to rapid panoptic staining, showing multinucleated epithelial cells, called Tzanck cells, suggesting viral inclusion by herpes virus (×400).

The treatment in 70% of the cases was carried out with acyclovir, followed by valacyclovir in 20% and foscarnet in 10%. Two female patients had the verrucous hypertrophic form, and the therapeutic approach in these cases was as described by several authors, with an antiviral combined with local therapy (surgical excision or topical imiquimod), with significant improvement (Fig. 4).^2^, ^3^

**Figure 4:**
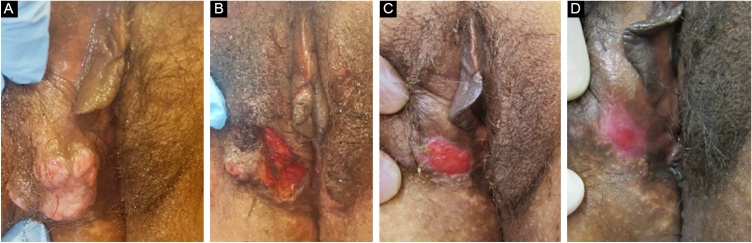
Patient with hypertrophic chronic mucocutaneous herpes tumor. **A**, Pre-treatment. **B**, In the immediate postoperative period after surgical removal of the tumor lesion. **C**, After 4 weeks of surgical excision, with the surgical wound undergoing healing. **D**, After 10 weeks of the procedure, with the healed lesion.

The recommended treatment for chronic ulcerated forms is the use of oral acyclovir, at a daily dose of 1,000 mg, initially for one to two weeks. This daily dose can be maintained or increased to 4,000 mg, and the medication can be administered intravenously (10 mg/kg 3×/day) in cases of resistance or to overcome bioavailability problems for the same period. If therapeutic failure persists, oral valacyclovir (daily dose of 3,000 mg) or famciclovir (daily dose of 550‒1,500 mg) is indicated for one to two weeks. If it is considered a resistant viral population, whose main associated factor is the prolonged use of antivirals, the use of antivirals with other mechanisms of action, such as foscarnet, cidofovir, and vidarabine, is suggested.^1^, ^3^, ^4^, ^8^ In addition to this targeted drug approach, aimed at a direct fight against HSV, it is essential to reverse the patient immunosuppression.

Chronic mucocutaneous herpes, despite being well established in the literature, is rare and little known. The diversity of clinical presentations and therapeutic resistance reinforces the challenge in the management of this disease. Thus, knowing the aspects detailed in the present report helps physicians in the diagnosis and management of the disease, optimizing therapy, and reducing patient morbidity.

## Financial support

None declared.

## Authors' contributions

Karina Baruel de Camargo Votto Calbucci: Drafting and editing of the manuscript; collection, analysis, and interpretation of data; critical review of the literature; critical review of the manuscript; approval of the final version of the manuscript.

John Verrinder Veasey: Design and planning of the study; effective participation in research orientation; intellectual participation in the propaedeutic and/or therapeutic conduct of the studied cases; drafting and editing of the manuscript; collection, analysis, and interpretation of data; critical review of the literature; critical review of the manuscript; approval of the final version of the manuscript.

## Conflicts of interest

None declared.

## References

[1] Barde C., Piguet V., Pechère M., Masouye I., Saurat J.H., Wunderli W. (2011). Management of resistant mucocutaneous herpes simplex infections in AIDS patients: a clinical and virological challenge. HIV Med..

[2] Siqueira S.M., Gonçalves B.B., Loss J.B., Estrella R.R. (2019). Vegetative chronic genital herpes with satisfactory response to imiquimod. An Bras Dermatol..

[3] Beutner K.R. (1992). Rational use of acyclovir in the treatment of mucocutaneous herpes simplex virus and varicella zoster virus infections. Semin Dermatol..

[4] Straus S.E., Smith H.A., Brickman C., Miranda P., McLaren C., Keeney R.E. (1982). Acyclovir for chronic mucocutaneous herpes simplex virus infection in immunosuppressed patients. Ann Intern Med..

[5] Whitley R.J., Roizman B. (2001). Herpes simplex virus infections. Lancet..

[6] Krusinski P.A. (1988). Treatment of mucocutaneous herpes simplex infections with acyclovir. J Am Acad Dermatol..

[7] Groves M.J. (2016). Genital herpes: a review. Am Fam Physician..

[8] Wauters O., Lebas E., Nikkels A.F. (2012). Chronic mucocutaneous herpes simplex virus and varicella zoster virus infections. J Am Acad Dermatol..

